# Plastic deformation of the forearm in adults: an analysis of 30 cases

**DOI:** 10.1186/s13018-014-0117-0

**Published:** 2014-12-02

**Authors:** Wu Tianhao, Liu Yueju, Zhang Yingze, Wu Xirui

**Affiliations:** Department of Traumatic Emergency Center, Third Hospital of Hebei Medical University, No. 139 Zi Qiang Road, Shijiazhuang, 050051 Hebei People’s Republic of China; Key Orthopedic Biomechanics Laboratory of Hebei Province, Shijiazhuang, 050051 People’s Republic of China

**Keywords:** Plastic deformation, Adult, Forearm fracture

## Abstract

**Background:**

Plastic deformation of the forearm is a rare and frequently missed injury in adults that can result in a significant loss of forearm rotation. The condition is reported mainly in Western countries; however, it is not uncommon in Eastern developing countries. We conducted a retrospective study of 30 cases of forearm deformation to find common factors to increase awareness of the condition in trauma doctors.

**Methods:**

We analyzed 30 cases of forearm plastic deformation in adult patients first diagnosed and treated at the Orthopedic Department of our hospital between January 2000 and June 2012. Patients’ age, injury mechanism, therapeutic process, and forearm rotation function were recorded for further analysis.

**Results:**

The average patient age was 21.3 years (range, 17–24 years), and the most common injury occurred at the right forearm in 29 patients (96.7%) when the arm became trapped in a machine with moving rollers. The remaining patient was injured while skiing. Twelve patients had a radial or ulnar fracture, 16 patients sustained no fracture, one patient had both radial and ulnar fractures, and one patient had an ipsilateral humeral fracture. Thirteen patients agreed to surgical osteotomy to reset the fracture or the distal/proximal radioulnar joint dislocation. All patients obtained good forearm function postoperatively, with an average pronation of 77° and supination of 78°. One patient refused surgical treatment, which led to forearm deformity and dysfunctional rotation.

**Conclusions:**

We found that adult patients with forearm plastic deformation had similar age (17–24 years) and injury mechanism (entrapment in moving rollers in machines). In cases where the resulting ulnar or radial fractures and the distal/proximal radioulnar dislocation cannot be reset, we advise surgical osteotomy.

## Background

Plastic deformation of the forearm is a rare and frequently missed injury in adults that can result in a significant loss of forearm rotation. To our knowledge, there are only ten previous case reports describing this injury from 1982 to the present, in Western countries [1-10]. However, the injury is becoming more common in China with increasing numbers of young men working with industrial machinery, suggesting that the injury is closely related to age and occupation. Our aim was to find common factors among patients to increase physicians’ awareness of this condition.

## Methods

### Patients

The orthopedic fracture registry at a single, level one trauma center was retrospectively reviewed for adult patients with forearm plastic deformation occurring between January 2000 and June 2012. The inclusion criteria were the following: adult patients with forearm plastic deformation with complete follow-up data and anatomical reduction of an ulnar or radial fracture. The exclusion criterion was incomplete epiphyseal closure. The study was exempted from an institutional review board approval, and the 30 patients met the inclusion criteria. The electronic medical record (including inpatient medical records, operative notes, outpatient medical records, and all radiographs) for each patient was reviewed by a single orthopedic surgeon (YL) who was not involved in the patients’ clinical care. Postoperative forearm rotation was assessed by another senior clinician (TW). Patients’ age, injury mechanism, therapeutic process, and forearm rotation function were recorded for further analysis.

### Surgical osteotomy

**Figure 1:**
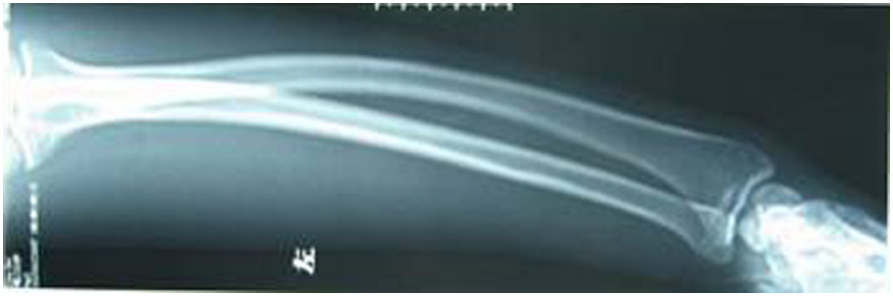
**X-ray of both ulnar and radial plastic deformation.**

**Figure 2:**
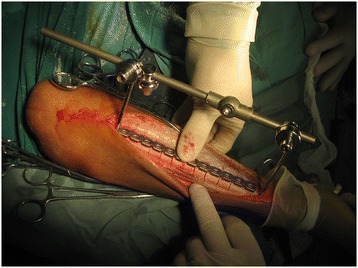
**Multiple-level osteotomies of the radius intraoperatively; an external fixator was used for temporary fixation.**

**Figure 3:**
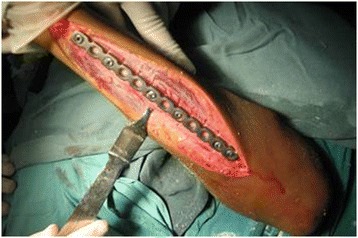
**Completed ulnar multiple-level osteotomies.**

**Figure 4:**
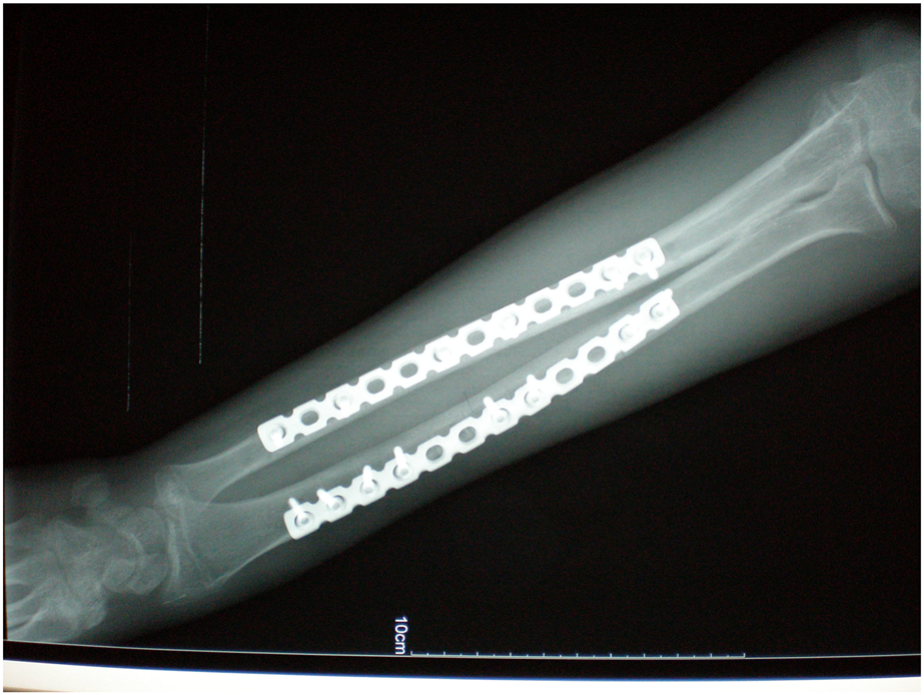
**X-ray showing perfect anatomic reduction and healed insufficiency fractures caused by the multiple-level osteotomies.**

Briefly, the surgical osteotomy was as follows: The radius and ulna were approached through separate incisions. The Thompson approach was used to expose the radius with the depth of the saw reaching the bone at approximately 2/3 of the bone diameter, followed by an external fixation for immediate and temporary osteotomy site compression (Figures 1 and 2). A 3.5-mm, AO/ASIF limited contact dynamic compression plate was then molded to fit the normal bow of the radius, and once all screws were placed, the external fixator was removed. Ulnar osteotomy followed the same method (Figure 3). Postoperative results included 70° of forearm supination and 80° pronation, with radiologically confirmed perfect anatomical reduction (Figure 4).

## Results

The average patient age was 21.3 years (range, 17–24 years) with the most common injury to the right forearm in 29 patients (96.7%) whose arm became trapped in a machine with moving rollers. The remaining patient was injured while skiing. Twelve patients had a radial or ulnar fracture, 16 patients sustained no fracture, one patient had both radial and ulnar fractures, and one patient had an ipsilateral humeral fracture. Thirteen patients agreed to surgical osteotomy to reset the fracture or the distal/proximal radioulnar dislocation. All patients obtained good forearm function postoperatively with 77° pronation and 78° supination. One patient refused surgical treatment, which led to forearm deformity and dysfunctional rotation (Table 1).

**Table 1:** **Date on patients**

**Patient number**	**Age/sex (years)**	**Mechanism**	**Fractures**	**Radioulnar dislocation**	**Osteotomy**	**Pronation/supination (degree)**
1	17/F	Skiing	SF	NO	NO	80/80
2	18/M	ERM	BF	DRD	YES	75/80
3	18/M	ERM	SF	NO	NO	85/90
4	18/M	ERM	SF	PRD	YES	80/90
5	25/M	ERM	NF	PRD	YES	70/75
6	19/M	ERM	SF	NO	NO	80/70
7	19/M	ERM	NF	NO	NO	80/80
8	24/M	ERM	SF	NO	YES	90/85
9	20/M	ERM	NF	NO	NO	80/80
10	23/M	ERM	NF	NO	NO	75/70
11	19/M	ERM	SF	NO	NO	80/75
12	19/M	ERM	NF	DRD	YES	70/75
13	19/M	ERM	SF	NO	NO	70/70
14	24/M	ERM	SF	DRD	YES	80/70
15	20/M	ERM	NF	NO	NO	70/70
16	22/M	ERM	SF	NO	NO	80/90
17	24/M	ERM	SF	DRD	YES	90/90
18	21/M	ERM	SF	NO	NO	85/80
19	19/M	ERM	NF	DRD	YES	80/70
20	18/M	ERM	NF	NO	NO	30/40
21	19/M	ERM	NF	NO	NO	70/75
22	20/M	ERM	NF	NO	YES	75/75
23	22/M	ERM	NF	NO	NO	80/75
24	23/M	ERM	NF	DRD	YES	85/80
25	22/M	ERM	NF	NO	YES	70/70
26	19/M	ERM	HF	NO	NO	65/80
27	21/M	ERM	NF	DRD	YES	70/80
28	21/M	ERM	NF	NO	NO	80/85
29	19/M	ERM	SF	DRD	YES	70/75
30	24/M	ERM	NF	NO	NO	75/75

*ERM* entrapment on moving rollers in machines, *HF* humeral fracture, *BF* both ulnar and radial fractures, *SF* single ulnar/radial fracture, *NF* no fractures, *DRD* distal radioulnar dislocation, *PRD* proximal radioulnar dislocation.

## Discussion

The primary mechanism of injury in our patients was accidental entrapment in moving machinery rollers. During a work with rotating machines, the patients’ hands became entangled in the conveyer belt, creating a bending pull to the forearm. The deforming force was continuous for several minutes, with sustained high force leading to either forearm fracture or dislocation at the radioulnar joint, depending on the length of time the force was sustained. Some patients sustained simultaneous humeral fractures because of violent upward forces [11]. Previous reports discuss forearm plastic deformation caused by others reasons such as skiing and road-traffic accidents [2,8]. But even in the published cases, entrapment in moving machinery rollers accounted for >99% of injuries. This cause of forearm plastic deformation is becoming more common in China, which is undergoing rapid mechanization, and we believe that the injury should be considered an occupational hazard with the need for an early prevention.

The second characteristic feature in our study was the age distribution, ranging from 17–25 years. Increasing bone maturation leads to greater mineralization, which hardens the collagen and hydroxyapatite complex, decreasing the bone’s flexibility [12]. This is a likely pathological basis for forearm plastic deformation in young adults. The typical clinical features were pain, diffuse swelling, gross deformity, tenderness, and restricted forearm rotation. Awareness of dislocation at the radioulnar joint is important to avoid misdiagnosis.

As with angulation in more typical forearm fractures in adults, plastic deformation can result in a loss of forearm pronation and supination, restoration of which is the goal of treatment. The treatment method varies in previous case reports. In our patients, closed reduction and above-elbow plaster of casting, maintained for 6–8 weeks, were the first recommendations. However, in patients with non-reducible ulnar or radial fractures or distal/proximal radioulnar dislocation, surgical osteotomy was recommended. Multiple-level osteotomies allow the length of the deformity to be addressed and can reduce the risk of nonunion, but in some cases, a single osteotomy was necessary because the diagnosis was initially missed, which resulted in marked intraoperative difficulty reducing the fracture or dislocation.

The limitations of this study include its retrospective nature and the lack of magnetic resonance imaging findings to detect the degree of forearm interosseous membrane injury, which provides greater detail regarding the mechanism of injury.

## Conclusions

In summary, adult patients with forearm plastic deformation had similar age and similar injury mechanism (entrapment in moving machine rollers). We advise surgical osteotomy when the associated ulnar or radial fracture and the distal/proximal radioulnar dislocation cannot be reset.
